# *ATRX* mRNA expression combined with *IDH1/2* mutational status and Ki-67 expression refines the molecular classification of astrocytic tumors: evidence from the whole transcriptome sequencing of 169 samples

**DOI:** 10.18632/oncotarget.1838

**Published:** 2014-03-21

**Authors:** Jinquan Cai, Pei Yang, Chuanbao Zhang, Wei Zhang, Yanwei Liu, Zhaoshi Bao, Xing Liu, Wenzhong Du, Hongjun Wang, Tao Jiang, Chuanlu Jiang

**Affiliations:** ^1^ Department of Neurosurgery, The Second Affiliated Hospital of Harbin Medical University, Harbin, China; ^2^ Beijing Neurosurgical Institute, Capital Medical University, Beijing, China; ^3^ Beijing Institute for Brain Disorders Brain Tumor Center; ^4^ Department of Neurosurgery, Beijing Tiantan Hospital, Capital Medical University, Beijing, China; ^5^ Chinese Glioma Cooperative Group (CGCG), China

**Keywords:** ATRX, IDH1/2, Ki-67, astrocytic tumors, whole transcriptome sequencing

## Abstract

Astrocytic tumors are the most common primary brain tumors in adults. *ATRX* mutations have been identified in gliomas and are correlated with its loss of expression, which causes alternative lengthening of telomeres (ALT) leading to genomic instability. In this study, we aimed to explore the role of *ATRX* mRNA expression alteration in the progression and subclassification of astrocytic tumors and examine its impact on clinical outcome. We investigated *ATRX* mRNA expression and its association with IDH1 and IDH2 mutations in 169 adult astrocytic tumors using whole transcriptome sequencing. In our cohort, low *ATRX* mRNA expression was detected in 68% of astrocytomas, 50% of anaplastic astrocytomas and 41.6% of glioblastomas. Low *ATRX* expression closely overlapped with mutations in *IDH1/2* (P<0.0001) in astrocytic tumors across WHO grades II–IV. Significant association between low *ATRX* expression and longer overall survival was identified in our cohort (P<0.01). *ATRX* combined with *IDH1/2* and Ki-67 was used to re-classify patients with astrocytic tumors: group A1 containing *IDH1/2* mutations and low *ATRX* expression predicted a better prognostic outcome, whereas group A3 carrying wild-type *IDH1/2* and high Ki-67 expression had the shortest overall survival; IDH-mutant tumors with low *ATRX* expression and IDH-wild-type tumors with high Ki-67 expression were grouped into group A2. In summary, our results showed that *ATRX* in cooperation with *IDH1/2* and Ki-67 defines three subgroups of astrocytic tumors regardless of the conventional WHO grades consensus. The molecular stratification in astrocytic tumors may aid in treatment strategy selection, therapeutic trial design, and clinical prognosis evaluation.

## INTRODUCTION

Glioma is the most common type of primary brain tumor among adults [1]. Tumors are graded on a WHO consensus-derived scale of I to IV according to their degree of malignancy, as judged by various histological features accompanied by genetic alterations [2]. Astrocytic tumors, which are the most common group of human gliomas, have an inherent tendency for recurrence and malignant progression, and usually cannot be cured by neurosurgical resection, radiotherapy and chemotherapy [3]. Malignant astrocytic tumors such as glioblastoma (GBM) are the most lethal intracranial tumors [4, 5]. The prognosis of astrocytic tumors depends on certain clinical factors, most notably age at diagnosis, clinical status as measured by the Karnofsky score, and extent of tumor resection, as well as the histological type, tumor grade and several molecular markers [3].

Over the past 30 years, recurrent chromosomal, cell biological, and genetic alterations have implicated a number of molecules in the different histological types and malignancy grades of astrocytic tumors [6]. Recently, various molecular markers have been reported to correlate with an improved group of adult gliomas. Somatic mutations in isocitrate dehydrogenase 1 and 2 (*IDH1/2*) were detected in approximately 80 % of diffuse and anaplastic astrocytomas as well as secondary GBMs. Tumors with *IDH1/2* mutations had distinctive genetic and clinical characteristics, and patients with such mutations had a better outcome than those with wild-type (WT) IDH [7, 8]. We have previously reported that high expression levels of Ki-67 protein, which is associated with the cell cycle, mitosis and cell division, predict a shorter survival time for patients with malignant gliomas [9, 10]. Although several articles have delineated the molecular classification of astrocytic tumors, a more convenient method would be available for clinical application and would arouse the interest of medical practitioners [6, 11].

However, the clinical significance of molecular parameters for the diagnostic and prognostic prediction of astrocytic tumors is still limited. Recently, mutation/loss of *ATRX* was identified as a potent biomarker in lower-grade gliomas and was associated with recurrent gliomas [12-14]. Loss of *ATRX*-DAXX (death-domain associated protein) function impairs the heterochromatic state of telomeres, leading to telomere destabilization and thereby facilitating the development of alternative lengthening of telomeres (ALT) [15]. Recent studies characterized the molecular landscape of gliomas to better understand their molecular pathogenesis and to identify molecular subgroups of these tumors and aid in their classification for clinical management [13, 16, 17]. Identification of *ATRX*, *CIC*, and *FUBP1* mutations refined the prognostic information provided by the known markers *IDH*, *TP53*, and 1p/19q loss of heterogeneity (LOH) [13, 18].

In this study, we sought to detect *ATRX* mRNA expression alterations that would enhance our understanding of the biology of astrocytic tumors and provide novel potential markers for prognosis. To achieve our goal, we applied RNA-seq to 169 astrocytic tumor samples in which three grades of distinct *ATRX* mRNA expression was demonstrated [19, 20]. Our approach highlighted the power of RNA-seq technology in identifying *ATRX* as a prognostic marker and characterizing three subgroups of astrocytic tumors (referred to as A1, A2 and A3).

## RESULTS

### Decreased *ATRX* mRNA expression was characteristically present in low-grade astrocytomas

**Figure 1:**
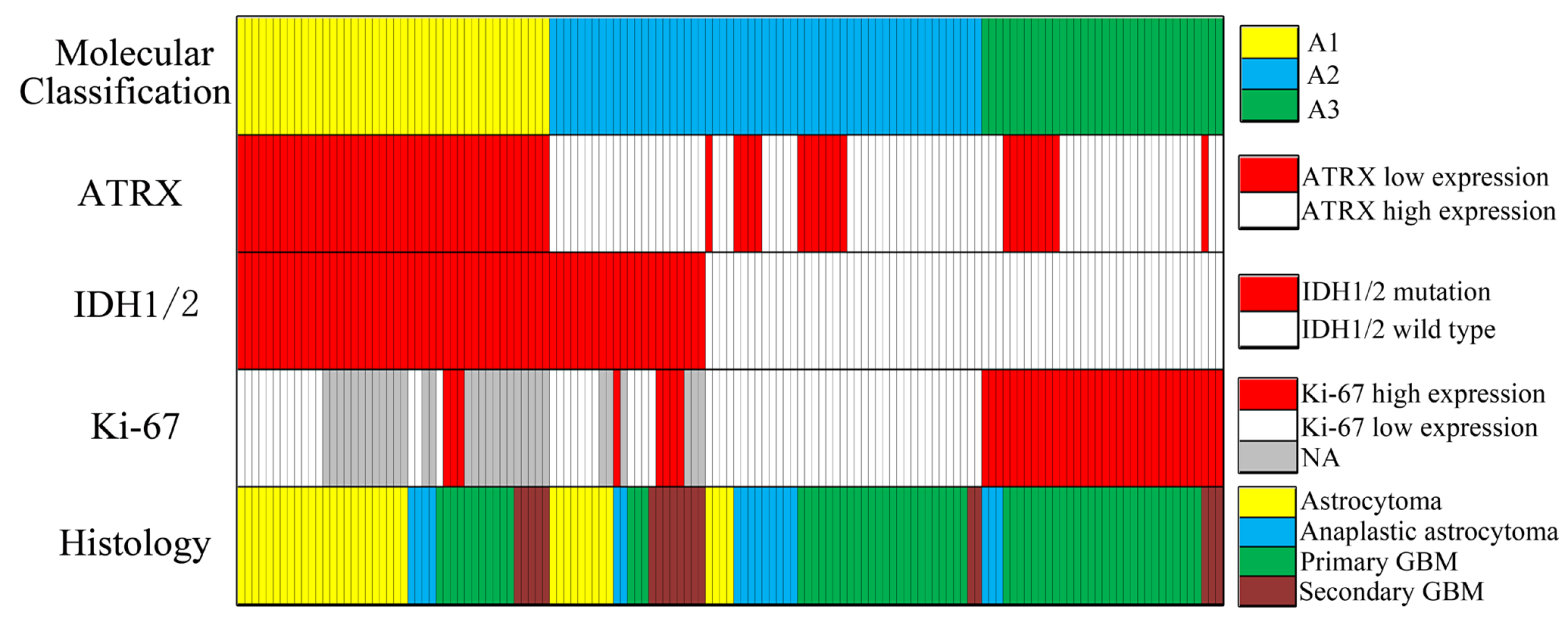
Overview of histology, molecular aberrations and molecular classification in the cohort (n=139) Each column represents a patient. “Molecular Classification” is defined as A1 (*IDH*-mut astrocytic tumors with *ATRX*-low; n=44), A2 (*IDH*-mut tumors with *ATRX*-high and *IDH*-WT with low Ki-67 expression; n=61), A3 (*IDH*-WT tumors with high Ki-67 expression; n=34).

**Figure 2:**
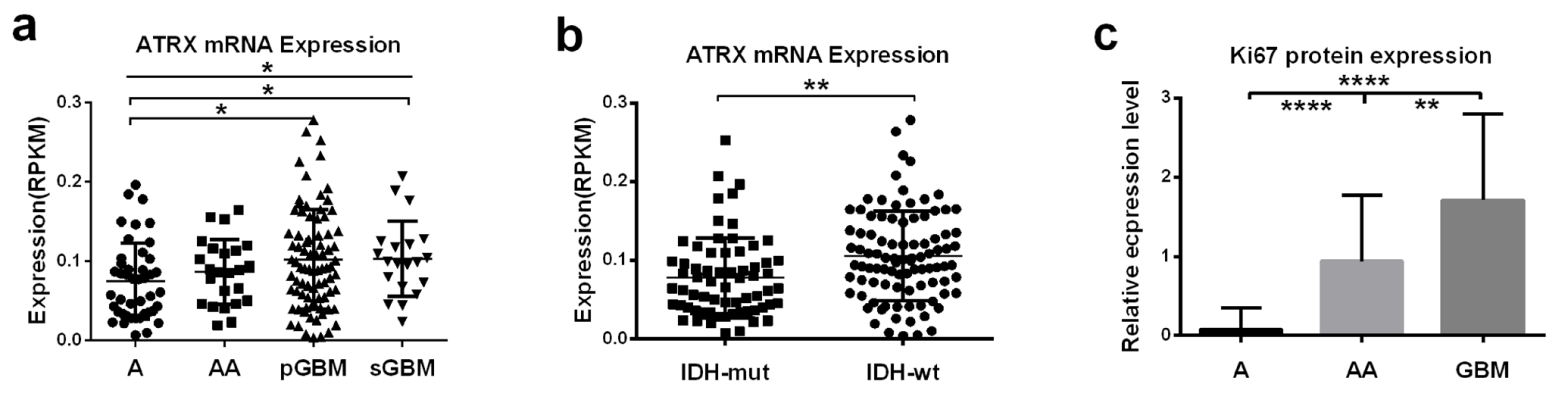
Correlation of *ATRX* mRNA expression and Ki-67 protein expression with histology and *IDH1/2* mutational status a. *ATRX* mRNA expression (RPKM) was different in grade II-IV astrocytic tumors (*P*<0.05) and decreased in As than in pGBMs and sGBM (*P*<0.05, *P*<0.05, respectively). b. *ATRX* mRNA expression (RPKM) in tumors with *IDH* mutations was decreased compared with *IDH*-wild-type tumors (*P*<0.01). c. Ki-67 protein expression significantly differed in the three grades of astrocytic tumors (*P*<0.0001) and the expression levels in GBMs was significantly higher than that in astrocytomas and anaplastic astrocytomas (*P*<0.0001 and *P*<0.01, respectively). *P < 0.05, **P < 0.01, ****P < 0.0001.

In our dataset, all *ATRX* RPKM scores were classified into two categories, with the median expression of them as cutoff point. There were 84 samples, harboring lower *ATRX* RPKM score than the cutoff point, in the *ATRX*-low group, which included 30 As, 12 AAs and 42 GBMs. The *ATRX*-high group was comprised of 14 As, 12 AAs and 59 GBMs which bored higher *ATRX* RPKM score than the cutoff point. The frequency of *ATRX* mRNA low expression was higher in As (30/44, 68%) than in AAs (12/24, 50%), in pGBMs (35/81, 43%) and in sGBMs (7/20, 33%) (Table 1, Fig.1; *P*<0.05, Chi-Square test).. Consistent with the analysis above, *ATRX* mRNA expression (RPKM) was different in grade II-IV astrocytic tumors (Fig.2a; *P*<0.05) and decreased in As than in pGBMs and sGBM (Fig.2a; *P*<0.05, *P*<0.05, respectively). These findings confirmed a strong correlation between *ATRX* mRNA expression and malignancy in astrocytic tumors, suggesting low *ATRX* mRNA expression was characteristically present in low-grade astrocytomas.

**Table 1:** Clinicopathological Characteristics of the patients

Varible		ATRX-Low (n=84)	ATRX-High (n=85)	p value
Median age		42	45	
Age	≥45	33	45	>0.05
	<45	51	40	
Gender	Male	43	63	<0.01
	Female	41	22	
Preoperative KPS score	≥80	43	42	>0.05
	<80	41	43	
Grade	II A	30	14	<0.05
	III AA	12	12	
	IV GBM	42	59	
	pGBM	35	46	
	sGBM	7	13	
IDH1/2 status	Mutation	44	22	<0.0001
	Wild type	34	60	
	NA	6	3	
Ki-67 expression	Low	31	39	>0.05
	High	14	31	
	NA	39	15	
Extent of surgery	Total	42	39	>0.05
	Subtotal	31	37	
	NA	11	9	
Radiotherapy	Yes	53	51	>0.05
	No	26	28	
	NA	5	6	
Chemotherapy	Yes	45	46	>0.05
	No	31	32	
	NA	8	7	

Abbreviations: A, astrocytoma; AA, anaplastic astrocytoma; KPS, Karnofsky;pGBM, primary GBM; sGBM, secondary GBM.

### *ATRX* mRNA expression alteration was strongly associated with *IDH1/2* mutations in astrocytic tumors

To investigate the association between *ATRX* mRNA expression alteration and *IDH1/2* mutations, we screened tumor samples in our cohort for *IDH1/2* mutations using pyrosequencing. In accord with previous reports, we identified *IDH1/2* mutations in 82.5% of As, 27.3% of AAs, 17.3% of pGBMs and 68.4% of sGBMs. Among the 84 tumor samples with low *ATRX* mRNA expression, 44 had mutations in either *IDH1* or *IDH2*, while 34 were WT for both genes, indicating a strong association between *ATRX* mRNA expression and *IDH1/2* mutations (Table 1, Fig.1; *P*<0.0001, Chi-Square test). In agreement with the analysis above, the expression of *ATRX* mRNA in tumors with *IDH* mutations was significantly decreased compared with WT *IDH* tumors (Fig. 2b; *P*<0.01).

### *ATRX* mRNA expression alteration was a potent prognostic factor and could subclassify astrocytic tumors in combination with *IDH1/2* mutations and Ki-67 expression

**Figure 3:**
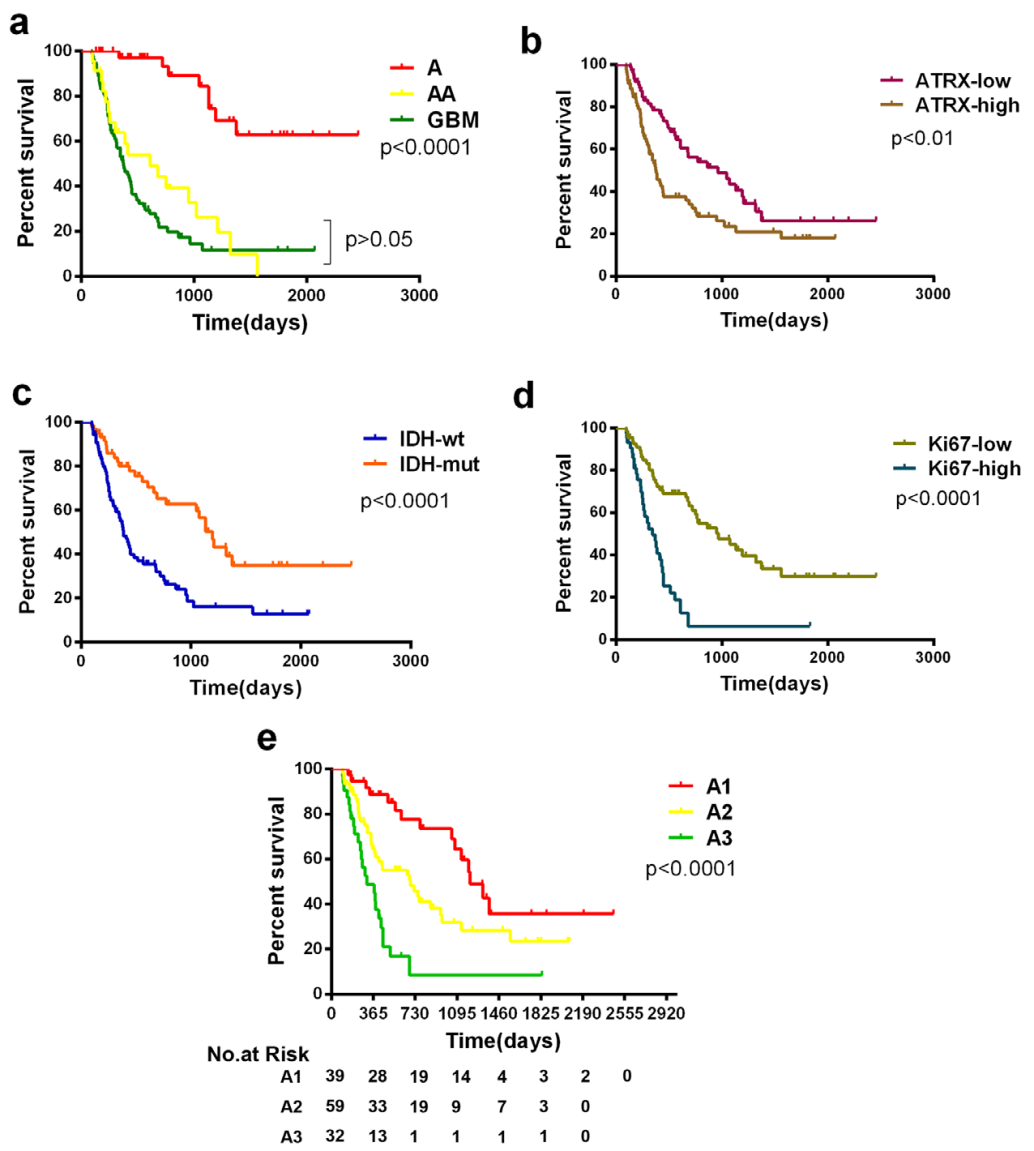
Kaplan-Meier estimates of survival for astrocytic tumor patients a. Among patients with astrocytic tumors of all grades, there was no significant difference in survival between anaplastic astrocytomas and GBMs when patients were only stratified by the histopathological diagnosis of their tumors. b, c, d. *ATRX*, *IDH1/2* status and Ki-67 are biomarkers associated with survival of patients in astrocytic tumors. e. Among all patients with astrocytic tumors (n=139, except non-classifiable), there was a significant difference in overall survival between the new three subgroups, and the A1 group and A2 group survived significantly longer (median OS, 1208 and 689 days, respectively) than the A3 group (309 days; *P*<0.0001).

**Figure 4:**
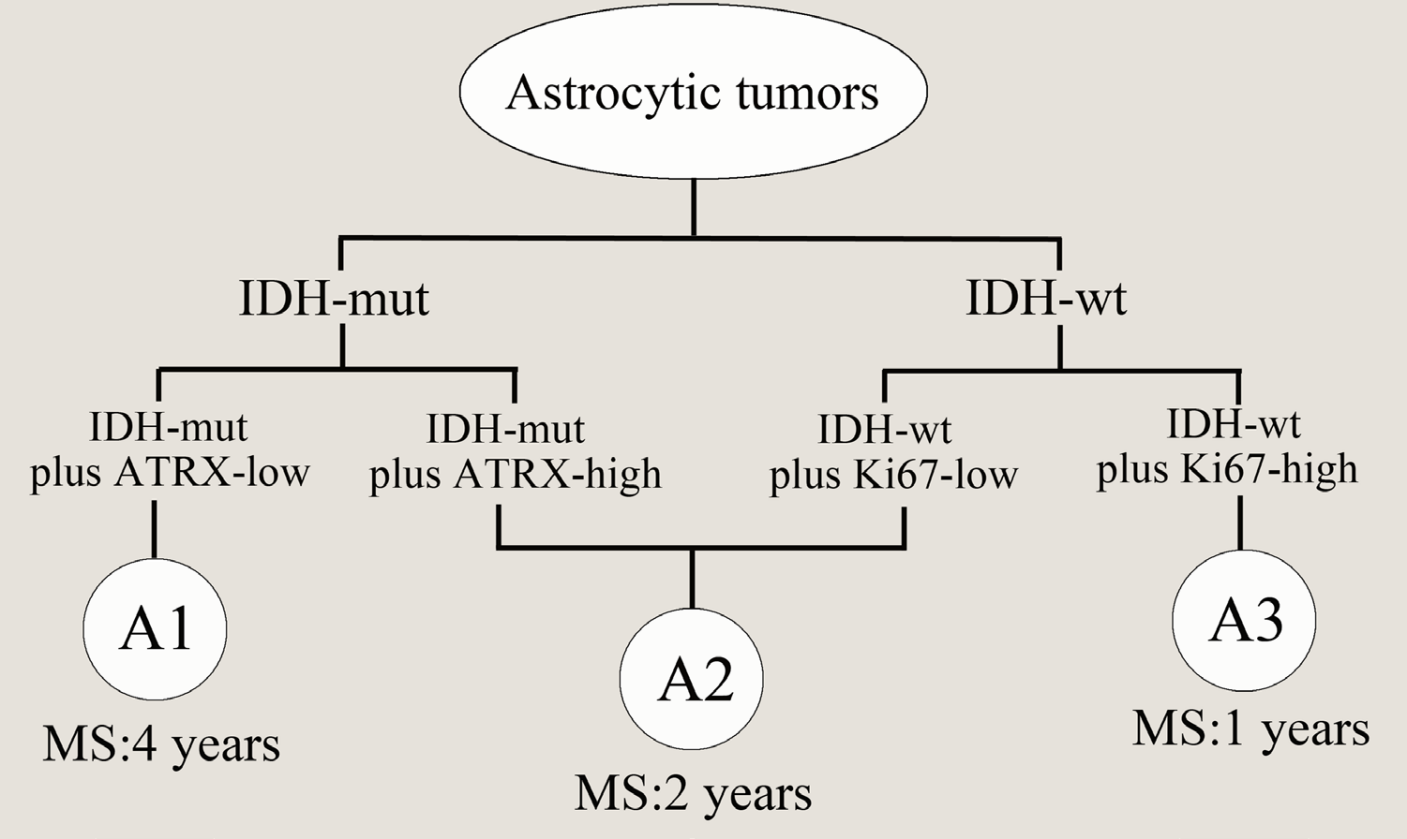
Model for classification of astrocytic tumors based on molecular markers *IDH*-mut tumors with *ATRX*-low was defined as A1 (median OS=4 years), *IDH*-WT tumors with high Ki-67 expression as A3 (median OS=1 years), and *IDH*-mut tumors with *ATRX*-high and *IDH*-WT with low Ki-67 expression were termed as A2 (median OS=2 years). MS: median OS.

The clinical characteristics of 169 patients in our cohort were described between the two groups with low or high *ATRX* mRNA expression levels (Table.1). Notably, in the present study, the prognostic value of histological grading was not significant in high-grade astrocytic tumors (Table 2, Fig.3a; AAs vs. GBMs: *P*>0.05). However, patients in the *ATRX*-low group displayed significantly longer overall survival than patients in the *ATRX*-high group (Table 2, Fig.3b; Median OS=965 vs. 381 days; log-rank test, *P*<0.01). Our results also illustrated that high Ki-67 expression was dominant in WT *IDH1/2* astrocytic tumors (Fig.1, *P*<0.05, Chi-Square test), and its relative expression level was significantly different in the three malignancy grades(Fig.2c). Therefore, to further refine the molecular classification of astrocytic tumors, we incorporated the other two prognostic markers into the model, *IDH1/2* mutational status and Ki-67 protein expression, which were also associated with the clinical outcome of patients with astrocytic tumors.(Fig.3c, Fig.3d, Table 2). Based on these findings, we first classified astrocytic tumors into *IDH*-mut and *IDH*-WT tumors and then defined *IDH*-mut tumors with *ATRX*-low as A1, *IDH*-WT tumors with high Ki-67 expression as A3, and grouped *IDH*-mut tumors with *ATRX*-high and *IDH*-WT tumors with low Ki-67 expression into A2 (Fig.4). The A1 subgroup was correlated with a better clinical outcome (median OS, 4 years). In contrast, the A3 subgroup was associated with a poorer clinical outcome (median OS, 1 year). Correlation of the A2 subgroup with respect to clinical outcome fell between the A1 and A3 subgroups (median OS, 2 years). As showed in Figure 3e, survival analysis of the new classification also demonstrated a remarkable separation of the clinical course in the three molecular subgroups (log-rank test, *P*<0.0001). To study the influence of the three molecular markers (IDH1/2, ATRX and Ki-67) we used in the our classidication schema, multivariate Cox regression analyses were used for the adjustment of these factors (Table 3). We confirmed that *IDH1/2* mutation status, *ATRX* mRNA expression and Ki-67 expression were three independent of each other prognostic factors in our cohort and the new classification scheme was dependent on these three factors to predict survival value. Furthermore, upon incorporation of only our classification and the WHO grading scheme (Table 3), the prognostic value of our classification was still significant, independent of the WHO grades, and served as an addition to the latter.

**Table 2:** Prognostic factors associated with OS in the univariate analysis for astrocytic tumors

Variable		Hazard ratio	95% CI	p value
Age	≥45 vs. <45	1.744	1.146-2.635	<0.01
Gender	Male vs. Female	0.756	0.486-1.177	>0.05
Preoperative KPS score	≥80 vs. <80	0.169	0.105-0.270	<0.0001
New Classfication	A1 vs. A2	2.103	1.143-3.868	<0.05
	A2 vs. A3	2.251	1.309-3.872	<0.01
Grade	A vs. AA	6.997	2.981-16.420	<0.0001
	AA vs. GBM	1.345	0.789-2.291	>0.05
IDH1/2 mutation status	Mut vs. Wt	2.552	1.596-4.082	<0.0001
ATRX mRNA expression	Low vs. High	1.872	1.228-2.855	<0.01
Ki-67 expression	Low vs. High	3.552	2.115-5.966	<0.0001
Extent of resection	Total vs. Subtotal	1.473	0.943-2.301	0.089
Radiotherapy	Yes vs. No	1.674	1.067-2.626	<0.05
Chemotherapy	Yes vs. No	0.791	0.504-1.240	>0.05

Abbreviations: OS,overall survival; A, astrocytoma; AA, anaplastic astrocytoma; KPS, Karnofsky performance status; Mut, mutation; Wt, wild type.

**Table 3:** Multivariate COX regression models for overall survival

Variable	Hazard ratio	95% CI	p value
COX model of Classification, IDH1/2, ATRX and Ki-67			
Classification(A1 vs. A2 vs. A3)	0.464	0.193-1.115	0.086
IDH1/2 (Mut vs. Wt)	2.884	1.137-7.313	0.026
ATRX (Low vs. High)	2.665	1.386-5.123	0.003
Ki-67 (Low vs. High)	5.626	2.337-13.545	<0.0001
COX model of Classification and Grade			
Classification(A1 vs. A2 vs. A3)	1.541	1.078-2.204	0.018
Grade (A vs. AA vs. GBM)	2.095	1.468-2.990	<0.0001

Abbreviations: A, astrocytoma; AA, anaplastic astrocytoma; Mut, mutation; Wt, wild type.

## DISCUSSION

Mutations and loss of expression of alpha-thalassemia/mental retardation syndrome X-linked (*ATRX*) was first reported in pancreatic neuroendocrine tumors [21]. The protein encoded by *ATRX* plays multiple cellular roles, including chromatin remodeling at telomeres where it is required for the incorporation of the histone variant H3.3 [15]. Additionally, multiple reports have demonstrated that *ATRX* mutation or loss of expression results in ALT and genomic instability [13, 15, 22], and many ALT cancers harbor mutations in *ATRX* or *DAXX* genes encoding proteins that interact with each other at telomeres [15, 21].

In several previous studies, *ATRX* mutation was reported to be strongly correlated with its loss of expression, and may predict better prognosis in astrocytic tumors with *IDH* mutations [13, 15, 21, 22]. However, whether *ATRX* mRNA expression alteration could affect overall survival of patients with astrocytic tumors remained unclear. In our present work, we first examined ATRX mRNA expression level using whole transcriptome sequencing, because RNA-seq can add benefits for gene expression analysis such as quantitation of transcripts, improved dynamic range, and additional capabilities for detecting expressed single nucleotide variants (SNVs), translocations, and transcript isoform switches compared with microarray and immunochemistry [27]; and from the dataset of 169 astrocytic tumors we found that patients with low *ATRX* expression had a significantly longer median overall survival than those with high *ATRX* expression (965 vs. 381 days). This suggests that *ATRX* mRNA expression alteration plays an important role in the clinical course of astrocytic tumors. This may further promote the development of clinical substratification for astrocytic tumors based on this molecular aberration. According to Jiao's study, a large frequency of *ATRX* mutation and loss was reported in grade II (67%) and III astroctyomas (73%) by using exon sequencing[13]. In Wiestler's report, the frequency of *ATRX* loss was 45% (29/65) in anaplastic astrocytomas, detected by immunohistochemistry, which was also much lower than that in Jiao's report (AA, 73%, 32/44)[28]. Another article delineated that the frequency of *ATRX* alteration, tested by both exon sequencing and immunohistochemistry, was 100% (4/4) and 53% (16/30) respectively in anaplastic astrocytomas[29]. In the present study, for the first time to our knowledge, we detected *ATRX* mRNA expression using whole transcriptome sequencing in a large sample size. 68% of grade II astrocytomas harbored low *ATRX* expression, similar to the Jiao's result from exon sequencing data, whereas the incidence of low *ATRX* expression was only 50% in anaplastic astocytomas and 33% in secondary glioblastomas, and up to 43% of primary glioblastomas contained low *ATRX* expression. We thought that the difference in the above articles mainly resulted from the number of samples and the different levels detected by different testing methods, both of which could impact the frequency of *ATRX* alteration.

*IDH1/2* mutations typically occur in low-grade gliomas and anaplastic astrocytic, oligodendroglial and mixed oligoastroglial tumors and in secondary GBMs, but rarely in primary GBMs [7, 30]. Numerous studies have identified *IDH1/2* mutations as a more powerful prognostic marker in glioma patients, and found that *IDH* mutational status delineates molecularly and clinically distinct subclasses of gliomas[31]. In 2009, Yan's study indicated that mutations of *IDH1* and *IDH2* occurred in majority of several types of malignant gliomas and reported the incidence of *IDH1/2* mutation in diffuse astrocytoma (90%), anaplastic astrocytoma (73%) primary GBM (5%) and secondary GBM (85%). In the present study, the frequency of *IDH1/2* gene mutations in astrocytoma, anaplastic astrocytoma, primary glioblastoma and secondary glioblastoma was 82.5% (33/40), 27.3% (6/22), 17.3% (14/81) and 68.4% (13/19), respectively. Our team previous reports delineated that the incidence of *IDH1/2* mutation in Chinese population with anaplastic astrocytomas and primary glioblastoma was separately 42.9% (24/56) and 16.1% (19/118) [32, 33] and the reasons why there was somewhat difference in the frequency of *IDH* mutations from the result of American and European reports might be explained as follows: (1) there was a much larger sample size in our study which could reveal much detailed overview of *IDH1/2* gene mutation in anaplastic astrocytomas; (2) the distinct proportion of histopathological subtypes; (3) the ethnic differences[7, 32-34]And recent articles have demonstrated that the *ATRX*, *IDH1/2* and *TP53* mutant phenotype is important in the early development and progression of astrocytic tumors [12, 13, 25, 28]. In our study, we also showed that decreased *ATRX* mRNA expression was characteristically enriched in low-grade astrocytomas and this *ATRX* alteration significantly overlapped with *IDH1/2* mutations (*P*<0.0001). Interestingly, the relationship between high Ki-67 expression levels and WT *IDH* was further confirmed in this study. Our previous reports showed that *IDH1* mutations were associated with low Ki-67 expression in primary GBMs [32], and that Ki-67 protein expression is an independent prognostic marker in GBM patients[10]. In contrast with *IDH1/2* mutations and decreased *ATRX* expression being widely considered as key aberrations in the early stage of astrocytic tumors [7, 35], increasing Ki-67 expression may be the final event in the progression of these tumors [9, 10, 33].

Thus, we speculated that *IDH1/2* mutations accompanied by *ATRX* or Ki-67 may represent a distinct biological process during the development of astrocytic tumors from the original tumor cells. Excitingly, regardless of histological grading, our new molecular classification model on the basis of *IDH1/2* mutational status and *ATRX*/Ki-67 expression could exactly reflect the biological properties of the three subgroups of astrocytic tumors with distinct clinical prognosis. A1 tumors are clearly defined with low *ATRX* expresion, and *IDH*-mut, likely representing low grade II astroctyomas with the best prognosis. A3 tumors mainly harbor *IDH*-wt, high Ki-67 expression with the poorest prognosis, like glioblastoma. A2 subgroup included patients with or without *IDH1/2* mutations, and also included cases with low and high *ATRX* expression. This phenomenon could be explained by tumor heterogeneity within this subgroup. As we all know, malignant gilomas are hardly composed of a single homogeneous population, but rather by a heterogeneous ensemble of cells that differ in many biological features, such as morphology, proliferation rate, invasive behavior, metastatic potential, drug resistance, gene expression and genetic abnormalities[36, 37]. The molecular basis of heterogeneity in gliomas was evidenced by previous studies that found markedly different genetic instability phenotypes from clinical specimens, and even intratumoral samples [17, 38-40]. A growing number of genetic aberrations and their frequency were identified in different types of human gliomas, some of which also displayed outstanding significance in molecular subclassification. Primary glioblastomas are characterized by *EGFR* amplification, *PTEN* mutation and absence of *IDH1/2* mutations, while secondary glioblastomas are characterized by *TP53* mutations, *IDH1/2* mutations and lack of *EGFR* amplification[41]. However, we didn't introduce these markers into our analyses because of the much lower frequency of *EGFR* amplification or *PTEN* mutation in our dataset than that from Western researches. Co-deletion of 1p/19q was recently shown to be associated with *CIC* and *FUBP1* mutations, mainly seen in oligodendroglioma and mutually exclusive with *ATRX* mutation [13, 42]. Integrating *IDH–CIC–FUBP1* has served as a good definition to characterize oligodendroglioma[13]. Thus, in our work, we analyzed neither LOH of 1p/19q nor the mutational status of *CIC/FUBP1* in astrocytomas. In 2013, *TERT* promoter mutations were reported in glioma, and the frequency of these mutations was particularly high among primary glioblastomas (65/78, 83%) and pure oligodendroglial tumors (35/45, 78%), while relatively low in astrocytomas, including grade II astrocytomas, grade III astrocytomas and secondary GBM (4/40, 11%), suggesting that primary GBMs with *IDH1/2*- wt and *TERT* promoter mutation could be a heterogenous subtype [23, 24, 26]. However, there was still controversies in the prognostic value of *TERT* promoter mutation[43]. This is the critical reason why we defined astrocytic tumors using *IDH1/2* mutational status plus *ATRX* or Ki67 expression, a common proliferation marker in clinical practice [10]. Undoubtedly, the introduction of additional genetic markers may help clear up some of the heterogeneity in A2 subgroup. As mentioned above, we still need to take advantage of classical and emerging biomarker to interpret the heterogeneity so that the molecular classification can further improve, providing new clue for prognosis, treatment selection.In conclusion, our results revealed that *ATRX* mRNA expression alteration plays a critical role in astrocytic tumors and a new molecular stratification can serve as a useful addition to conventional glioma classification. Compared with Jiao's classification[13], our scheme further classified three subtypes (referred as A1, A2 and A3) of I-A and I-X gliomas. A1 tumor was similar to I-A glioma, and A3 tumor was a branch of I-X glioma; A2 tumor was the transformation stage between A1 and A3. Anyway, new molecular stratification above could serve as a useful addition to conventional glioma classification.

## METHODS

### Clinical characteristics of samples

One hundred sixty-nine samples from the Chinese Glioma Genome Atlas (CGGA) were included in our study, including 44 astrocytomas (WHO II, As), 24 anaplastic astrocytomas (WHO III, AAs), 81 primary glioblastomas (WHO IV, pGBMs) and 20 secondary glioblastomas (WHO IV, sGBMs). All of the patients (age range: 18–81 years) underwent surgical resection. Patients were eligible for the study if their diagnosis was established histologically by two neuropathologists according to the 2007 WHO classification guidelines [2]. Tumor tissue samples were obtained by surgical resection. All patients provided written informed consent, and the study was approved by the ethics committees of the participating hospitals. Survival data were collected by clinics during patient visits and/or phone interviews. Patients who underwent biopsy alone were not followed up at our center and were therefore excluded from the survival analysis.

### Whole transcriptome sequencing

Total RNA was isolated using a RNeasy Mini Kit (Qiagen) according to the manufacturer's instructions. A pestle and a QIAshredder (Qiagen) were used to disrupt and homogenize frozen tissue. RNA intensity was checked using 2100 Bioanalyzer (Agilent Technologies) and only high quality samples with an RNA Integrity Number (RIN) value greater than or equal to 7.0 were used to construct the sequencing library. The subsequent steps included end repair, adapter ligation, size selection and polymerase chain reaction enrichment. The length of DNA fragment was measured using a 2100 Bioanalyzer, with median insert sizes of 200 nucleotides. The libraries were sequenced on the Illumina HiSeq 2000 platform using the 101-bp pair-end sequencing strategy. Short sequence reads were aligned to the human reference genome (Hg 19 Refseq) using the Burrows-Wheeler Aligner (BWA, Version 0.6.2-r126) [44].

### Molecular analyses

**IDH mutations**. Genomic DNA was extracted from frozen tissues with a QIAamp DNA Mini Kit (Qiagen) according to the manufacturer's protocol. DNA concentration and quality were measured using a Nano-Drop ND-1000 spectrophotometer (NanoDrop Technologies, Houston, TX). Pyrosequencing of *IDH1/2* mutations was supported by Gene-tech (Shanghai, China) and performed on a Pyro-Mark Q96 ID System (Qiagen, Valencia, Calif). The primers 5'-GCTTGTGAGTGGATGGGTAAAAC-3', 5'-Biotin-TTGCC AACATGACTTACTTGATC- 3' for *IDH1* and 5'-ATCCTGGGGGGGACTGTCTT-3', 5'- Biotin-CTCTCCACCCTGGCCT ACCT-3' for *IDH2* were used for PCR amplification, and the primers 5'-TG GA TGGGT AAAACCT-3' for *IDH1* and 5'-AGCCCATCACCATTG-3' for *IDH2* were used for pyrosequencing [32, 33].

**Ki-67 protein expression**. Immunohistochemistry was performed to detect Ki-67 protein expression according to the manufacturer's protocol. Anti-Ki-67 antibody (Santa Cruz Biotechnology, Santa Cruz, CA) was used at a dilution of 1:100. Each slide stained was individually reviewed and scored by two independent neuropathologists. Staining was scored using a 4-point scale from 0 to 3, with 0=no or rare occurrence of staining, 1 = 10% of cells positively stained, 2 = 10–30 % of cells positively stained, 3 = >30 % of cells positively stained. Score 2 and 3 were defined as strong nuclear staining in at least 10 % of the tumor cells. Score 0 and 1 were defined as positive staining of <30 % of cells. Controls without primary antibody and positive control tissues were included in all experiments to ensure the quality of the staining [9, 34].

### Statistical analysis

RNA sequencing data was downloaded from CGGA [1, 45]. Gene expression was calculated using the RPKM method (reads per kilobase transcriptome per million reads) [46, 47]. The RPKM method is able to remove the influence of varying gene lengths and sequencing discrepancies from the calculation of gene expression. Therefore, the calculated gene expression can be directly used to compare the differences in gene expression among samples. We defined the median value of *ATRX* mRNA expression RPKM score as the cutoff point in distinguishing the expression level of each sample. Certain a sample, harboring higher *ATRX* RPKM score than the cutoff point, fell into *ATRX*-high, and in contrast, certain a sample with lower *ATRX* RPKM than the cutoff was termed as *ATRX*-low.

Overall survival was estimated from the date of diagnosis to the date of either death or last follow-up. Patients were censored at the time they were last known to be alive. Overall survival was assessed using the Kaplan–Meier method and the log-rank test was used for comparison between groups. Student's t-test and analysis of variance (ANOVA) were used to determine significant differences in GraphPad Prism Version 6.01. Clinical and pathological characteristics between cohorts were compared using the Chi-Square test in SPSS, version 13.0, software for Windows (SPSS). All differences were considered statistically significant at *P*<0.05.
